# Supplementary material for: Measurement properties of the one-minute sit-to-stand test in children and adolescents with cystic fibrosis: A multicenter randomized cross-over trial
Source: PLoS One. 2021 Feb 12;16(2):e0246781. doi: 10.1371/journal.pone.0246781 (PMC7880481; doi:10.1371/journal.pone.0246781)
Supplement: S1 Table — (DOCX) [file pone.0246781.s001.docx]

| Outcomes | STST | 6MWT |
| --- | --- | --- |
| *Lung Function* |  |  |
| FVC (L) | 0.15 | 0.59^§^ |
| FVC (% predicted value) | 0.22 | 0.01 |
| FEV1 (L) | 0.20 | 0.56^§^ |
| FEV1 (% predicted value) | 0.27 | 0.03 |
| FEV1/FVC (%) | 0.09 | -0.14 |
| PEF (L/min) | 0.23 | 0.58^§^ |
| PEF (% predicted value) | 0.20 | 0.13 |
|  |  |  |
| *Muscle Strength* |  |  |
| MIP (cmH2O) | 0.31 | 0.48^§^ |
| MEP (cmH2O) | 0.32 | 0.54^§^ |
| QS (N) | -0.01 | 0.46^§^ |
|  |  |  |
| *CF-related medical history during previous year* |  |  |
| Exacerbations within last year | 0.16 | 0.11 |
| Hospitalisations within last year | -0.02 | 0.01 |

**S1 Table. Correlations between functional exercise capacity measured with the STST or the 6MWT and other clinically meaningful outcomes in 36 children with CF**

FEV1: forced expiratory volume in 1 second; FVC: forced vital capacity; MEP: maximal expiratory pressure; MIP: maximal inspiratory pressure; PEF: peak expiratory flow; QS: quadriceps strength; STST: sit-to-stand test; 6MWT: six-minute walking test.

Pearson or Spearman correlation coefficients according to the distribution of the variables. * p<0.05; ^§^ p<0.01
