# Supplementary material for: Measurement properties of the one-minute sit-to-stand test in children and adolescents with cystic fibrosis: A multicenter randomized cross-over trial
Source: PLoS One. 2021 Feb 12;16(2):e0246781. doi: 10.1371/journal.pone.0246781 (PMC7880481; doi:10.1371/journal.pone.0246781)
Supplement: S2 Table — (DOCX) [file pone.0246781.s002.docx]

| HRQOL Outcomes | STST | 6MWT |
| --- | --- | --- |
| *Child-reported outcomes* |  |  |
| Physical functioning | 0.29 | 0.38* |
| Vitality | 0.45 | 0.56* |
| Emotional functioning | 0.19 | 0.17 |
| Eating problems | 0.01 | -0.06 |
| Treatment burden | -0.03 | -0.19 |
| Health perceptions | -0.22 | 0.04 |
| Social functioning | 0.37* | 0.42* |
| Body image | -0.01 | 0.04 |
| Role functioning | 0.01 | -0.05 |
| Weight | 0.33 | 0.52 |
| Respiratory symptoms | -0.26 | 0.04 |
| Digestive symptoms | 0.09 | 0.10 |
| Total score | 0.15 | 0.26 |
| *Parent-reported outcomes* |  |  |
| Physical functioning | 0.38 | -0.02 |
| Vitality | 0.05 | -0.30 |
| Emotional functioning | 0.30 | -0.17 |
| School performance | 0.21 | -0.02 |
| Eating problems | -0.20 | 0.01 |
| Treatment burden | 0.01 | 0.05 |
| Body image | 0.19 | 0.09 |
| Health perceptions | 0.01 | -0.17 |
| Weight | -0.16 | 0.20 |
| Respiratory symptoms | -0.37 | -0.39 |
| Digestive symptoms | -0.16 | -0.39* |
| Total score | 0.03 | -0.04 |

**S2 Table. Correlations between functional exercise capacity measured with the STST or the 6MWT and the health-related quality of life questionnaire (HRQOL) responses as provided by the children with CF and by one of their parents for children under 14**

HRQOL: health-related quality of life; STST: sit-to-stand test; 6MWT: six-minute walking test. Pearson or Spearman correlation coefficients according to the distribution of the variables. * p<0.05
