# Supplementary material for: Measurement properties of the one-minute sit-to-stand test in children and adolescents with cystic fibrosis: A multicenter randomized cross-over trial
Source: PLoS One. 2021 Feb 12;16(2):e0246781. doi: 10.1371/journal.pone.0246781 (PMC7880481; doi:10.1371/journal.pone.0246781)
Supplement: S3 File — (DOCX) [file pone.0246781.s005.docx]

**Trial Protocol Exercise field tests in Cystic Fibrosis:**

**ID-RCB Number: 2016-A01377-44**

**CNIL Number: 2021977 v 0**

**PROJECT TITLE**

Comparison between the one-minute sit-to-stand test (STST) and the 6-minute walking test (6MWT) in children and adolescents with Cystic Fibrosis (CF)

**PROMOTOR**

Pediatric Department (CF Unit), Le Havre Hospital, F-76600, Le Havre, driven by Dr. Pascal Le Roux, MD.

**PRINCIPAL INVESTIGATORS**

Pascal Le Roux, MD, Pediatric Department (CF Unit), Le Havre Hospital, F-76600, Le Havre.

pascal.leroux@ch-havre.fr

Yann Combret, PT, MSc, Pediatric Department (CF Unit), Le Havre Hospital, F-76600, Le Havre.

yann.combret@gmail.com

Charlotte Gennari, PT, Cystic Fibrosis Center, Caen University Hospital, F-14003, Caen.

cha.gennari@gmail.com

Fairuz Boujibar, PT, Rouen University Hospital, F-76000, Rouen

f.boujibar@gmail.com

**Study question**

Is the STST a valid tool to measure functional exercise capacity; do STST performance correlates with the distance walked during the 6MWT in children and adolescents with CF?

**Research Location**

Pediatric Department (CF Unit), Le Havre Hospital, F-76600, Le Havre.

CF Center, Caen University Hospital, F-14003 Caen.

CF Center, Rouen University Hospital, F-76000 Rouen.

**RESEARCH OBJECTIVES**

**General objectives**

To assess the validity of the STST to measure functional exercise capacity in children and adolescents with CF; and to address the associations between STST performance and other meaningful outcomes (respiratory and peripheral muscle strength, health-related quality of life, cardio-respiratory responses during both tests).

**Specifical objectives**

Main objective:

Assess the correlation between the STST performance and the distance walked during the 6MWT

Secondary objectives:

- Address the correlations between STST and 6MWT performances and the cardio-respiratory responses during both tests (heart rate HR; respiratory rate RR; pulsed oxygen saturation SpO2; blood pressure BP; dyspnea and leg fatigue)
- Address the correlations between both tests’ performances and; pulmonary function (forced vital capacity FVC; forced expiratory volume in 1s FEV1; peak expiratory flow PEF), respiratory muscle strength (maximal inspiratory and expiratory pressures MIP and MEP), quadriceps strength (QS) and health-related quality of life (HRQOL)
- Address the correlations between both tests’ performances and; anthropometric data (age, height, weight, body mass index BMI), hospitalizations and exacerbations within last year
- Question the specific insights of the STST in young children (from 6 to 10 years of age)
- Compare the cardio-respiratory responses between both similar tests (HR, RR, SpO2, BP, dyspnea and leg fatigue)
- Compare the cardio-respiratory responses between both different tests (HR, RR, SpO2, BP, dyspnea and leg fatigue)

**Data collection**

At the arrival of the subjects, investigators will collect:

- Age
- Gender
- Weight
- Height
- BMI
- Medical treatments
- Number of hospitalizations last year
- Number of exacerbations last year
- Lung function (FEV1, FVC, FEV1/FVC, PEF)
- Respiratory muscle strength (MIP and MEP°
- QS
- HRQOL
- Subjects signed consent

During the study procedure, investigators will collect:

- STST performances
- Distance walked during the 6MWT
- 6MWWork (Distance * weight)
- HR
- RR
- SpO2
- BP
- Dyspnea
- Leg fatigue

**STUDY HYPOTHESIS**

Investigators hypothesize that the STST is a valid tool and that STST performance will correlate with the distance walked during the 6MWT in children and adolescents with CF

**VALIITY OF THE STUDY**

External validity:

The aim of this study is to address the validity of the STST. This study also aims to compare cardio-respiratory responses during the STST and the 6MWT and to evaluate the associations between the STST and clinically important outcomes.

If the STST is valid, its use could be generalized in exercise testing for children and adolescents with CF. Moreover, this test could induce lower cardio-respiratory adaptations, dyspnea and fatigue and could easily be used in young children with CF. This test could be a reasonable option for addressing functional exercise capacity in young individuals with CF.

Further research could therefore be conducted to assess the precise repeatability of the STST as well as its responsiveness through the MCID (Minimal Clinically Important Difference).

Internal validity:

Selection bias: The same inclusion criteria will apply in the different CF Centers. All the subjects included will be free from exacerbation for at least 4 weeks to be included. This will be defined as the absence of a hospitalization or an intravenous antibiotic cure for at least 4 weeks [1]. All the analyses will be conducted on a single homogenous population of children and adolescents with CF.

Observation bias: All the procedures of the study will be similar in each of the CF Center. Field tests will be conducted according to the latest American Thoracic Society (ATS) and European Respiratory Society (ERS) guidelines [2]. The same rest period between each tests of 30 minutes will apply in each CF Centers [2]. This will minimize the effects of each testing procedures on the others. In order to account for the learning effect described for both 6MWT and STST, each test will be conducted twice [2, 3]. Standardized verbal information will be provided before the 6MWT in each of the CF Centers [2]. Although there is no mention of standardized information for the STST in the literature, we aimed to standardized the explanations before the test in all the Centers (see below). All the tests will be conducted within the same day. Subjects should not have had eaten 2 hours before the tests. The order of the tests will be randomized.

Confusion bias: This study is a randomized cross-over trial. Both tests will be conducted with the same subjects. In order to minimize the variation between subjects, 1 or 2 investigators maximum will conduct the tests in each center. The results of the first STST and 6MWT will not be communicated to the subjects before the second row to avoid any external influence.

**STUDY POPULATION**

**Inclusion criteria:**

- Subjects with CF;
- Aged 6 to 18 years;
- Male of female;
- Signed informed consent by the children and their parents, 24-hour pre-inclusion delay;
- Routinely assessed in Le Havre, Rouen or Caen CF Center
- Clinically stable (no exacerbation for at least 4 weeks)

**Non-inclusion criteria:**

- Contra-indications to exercise testing;
- Musculoskeletal or neurological disorders avoiding the realization of the field tests (fracture, sprain, peripheral or central nervous disease…);
- Other respiratory restrictive (scoliosis …) or obstructive (asthma…) disease;
- Other disease avoiding the realization of the tests;
- Unability to understand the study procedures;
- Symptoms of beginning exacerbation (fever, mucus plugging …)

**Exclusion criteria:**

- Unability to complete the study procedure
- Appearance of a non-inclusion criteria

**Sample size estimation**

The sample size estimation showed that 36 participants were necessary to detect a correlation coefficient of 0.45 between the STST and 6MWT performances, with 80% power and an alpha level of 0.05. The final sample size aimed for was 40 since it was estimated that there would be an attrition rate of approximately 10%, based on experience in using these clinical tests with children: the 2x2 STST and 6MWT protocols together, for example, lasted for at least 2 hours and had to be completed within the same day. Reasonable allowance was also made for unexpected factors such as the inability of a participant to complete all of the tests or the withdrawal of consent following inclusion in the study.

**Randomization procedure**

The randomization sequence will be computer-generated by a person not involved in the study using a random block size on a 1:1 ratio and will be sent to each of the CF Centers at the beginning of the study by the principal investigator (YC).

**Study procedures**

Data collection

After the reception of the signed informed consent, the investigators will collect the age, gender, weight, height, BMI, number of exacerbations and hospitalizations in the last year and the results of the latest pulmonary function testing (FVC, FEV1, PEF).

Respiratory muscle strength will then be measured. MIP and MEP will be measured using a unidirectional valve electronical manometer MicroRPM PUMA© according to the American Thoracic Society latest guidelines [4]. Each measure will be conducted three times and the best value will be recorded. The data collection of respiratory muscle strength will be the same in each CF Center.

QS will be measured using a hand-held dynamometer. Computerized dynamometric assessment of the quadriceps muscle is reproducible in adolescents with CF [5]. However, due to financial and technical limitations, we will be using a hand-held dynamometer. QS will be measured using the MicroFET2© that has shown reliability in both healthy adolescents and patients with COPD [6, 7]. High inter-rater reliability has been proven to ensure reproducible values between each CF Center. Standardized measures will be conducted using a belt stabilization to avoid measures errors [6, 7]. Each child will be seated with the legs pending, hip and knee flexed at 90° and the dynamometer will be placed on the anterior face of the dominant leg. 5 repetitions (lasting from 4 to 6 seconds) will be conducted and the best of the last 3 values will be recorded for the analysis. A rest period of 30 to 60 seconds will be respected between each measure [6].

All the children will complete the CFQ-R (Cystic Fibrosis Questionnaire). The French version of this scale has been validated [8]. 3 different questionnaires have been described: for children from 6 to 11 years, from 12 to 13 years and older than 14 years. All the procedures for the children and the investigators are precisely described within the scale completion document. The investigators will only have to help the children from 6 to 11 years by reading the questions and give examples whenever required. All the questions will be verbally answered by the children and answers will be reported on the questionnaire by the investigators. Two combinations are described for the different questions: « Très vrai / Vrai dans l’ensemble / Un peu vrai / Pas du tout vrai » and « Toujours / Souvent / parfois / Jamais ». A parental version of the questionnaire will be completed by the parents for the children younger than 14. Older children will complete the questionnaire on their own. The questionnaire will be filled in a quiet room.

Field tests realization:

Both field tests will be conducted twice. After the primary data collection, each test will be conducted once to account for the learning effect. The results of these first tests will not be transmitted to the children.

Both tests will then take place a second time. A 30-minute rest period will last between the habituation tests and the first field test and between both tests. The order of these tests will be randomized. HR, RR, BP, SpO2, dyspnea and leg fatigue will be measured before and after each test.

*STST*

STST will be conducted in a quiet room, in a similar environment in each center. The test will be performed using a vertically adjustable chair without arm-rests to account for the variability in leg length in participants. The chair height will be set to ensure 90° of knee flexion during the test. The test will be explained using standardized information in all Centers, and subjects will be allowed to perform several sit-to-stand movements before the test to familiarize themselves with the procedure. Beginning position will be subjects seated, with a 90° knee flexion angle and arms across the chest [10]. Explanations will be: « This test aims to assess your exercise capacity. The aim is to stand up as many times as possible in 1-minute. The number of repetitions that you will perform will be recorded. I will let you know when there will be only 15 seconds left. You are allowed to stop or rest during the test ». Resting periods will be authorized but the clock will not be stopped if the subject stops.

*6MWT*

The 6MWT will be conducted according to the ATS/ERS guidelines [2]. The test conditions will be standardized in each Center. The subjects will be asked to walk the greatest distance they can reach in 6-minutes. The test will take place in a 30-meters corridor. A visible mark will be placed at both extremities. Standardized information will be given each minute according to guidelines « Keep going» as well as the time remaining before the end of the test. Resting periods will be authorized but the clock will not be stopped if the subject stops.

*Rest periods*

A 30-minutes rest period will take place between the first and the second row of field tests. Similarly, a 30-minutes rest period will also take place between each different test. The rest period will take place in a quiet environment. The subjects will be asked to stay calm, not perform any exercise, and a cold drink (water but not sweetened drink) will be proposed.

Study design:

Children with CF meeting the inclusion criteria

*24-hour pre-inclusion delay*

Signed informed consent

Data collection

Randomization

6MWT

STST

STST

6MWT

Data collection

This study will be conducted according to the protocol and the legislation for clinical trial conduction.

**Research plan**

Study start date: September 2017

Duration of the study: 2 years

**Analysis**

Statistical analysis will be conducted with GraphPad Prism 5. The distribution of the variables will be evaluated with the Shapiro-Wilk test and parametric or non-parametric tests will be utilized accordingly. Pearson and Spearman tests will be conducted to assess the correlations between the different outcomes.

The significance level will be set at p<0.05.

**Stopping criteria**

All the investigators of this study are habituated to the conduction of such field tests and will consistently be watching over the children included in each Center. No side effects will be expected to occur.

The study procedure will be stopped if any exclusion criteria appears or upon request of the children.

**Data accessibility:**

The investigators will be the only person allowed to manipulate the data collected.

Children and their relatives will have access of their own data upon request at any time.

**Data conservation:**

All the data collected will be sent to Mr. Yann Combret, principal investigator, at the Le Havre Center after inclusion completion in each Center.

Data will be kept in a locked particular closet, inside the Le Havre Center.

**Final rapport:**

This study is aimed to be published in English in a peer-review journal indexed in MEDLINE/Pubmed.

**REFERENCES**

[1] O’Neill K, Tunney MM, Johnston E, Rowan S, Downey DG, Rendall J et al. Lung clearance index in adults and children with cystic fibrosis. *Chest*. 2016; [EPub] DOI 10.1016 /j. chest.2016.06.029.

[2] Holland AE, Spruit MA, Troosters T, Puhan MA, Pepin V, Saey D et al. An official European Respiratory Society/American Thoracic Society technical standard: field walking tests in chronic respiratory disease. *Eur Respir J*. 2014; 44: 1428-1446.

[3] Ziegler B, Rovedder PME, Oliveira CL, Silva FA, Dalcin PTR. Repeatability of the 6-minute Walk test in adolescents and adults with cystic fibrosis. *Respir care*. 2010; 55(8): 1020-1025.

[4] American Thoracic Society/European Respiratory Society. ATS/ERS Statement on respiratory muscle testing. *Am J Respir Crit Care Med*. 2002; 166(4): 518-624.

[5] Hussey J, Gormley J, Leen G, Greally G. Peripheral muscle strength in young males with cystic fibrosis. *J Cyst Fibros*. 2002; 1(3): 116-21.

[6] Mentiplay BF, Perraton LG, Bower KJ, Adair B, Pua YH, Williams GP et al. Assessment of lower limb muscle strength and power using hand-held and fixed dynamometry: a reliability and validity study. *PLoS One*. 2015; 10(10): e0140822.

[7] Bachasson D, Villiot-Danger E, Verges S, Hayot M, Perez T, Chambellan A et al. [Maximal isometric voluntary quadriceps strength assessment in COPD]. *Rev Mal Respir.* 2014; 31(8): 765-70.

[8] Bohannon RW, Kindig J, Sabo G. Isometric knee extension force measured using a hand-held dynamometer with or without belt stabilization. *Physiother Theory Pract*. 2012; 28: 562-8.

[9] Henry B, Aussage P, Grosskopf C, Goehrs JM. Development of the Cystic Fibrosis Questionnaire (CFQ) for assessing quality of life in pediatric and adult patients. *Quality of life research*. 2003; 12: 63-76.

[10] Radtke T, Puhan MA, Hebestreit H, Kriemler S. The 1-min sit-to-stand test – A simple functional capacity test in cystic fibrosis? *J Cyst Fibros*. 2016; 15(2): 223-6.
