# Supplementary material for: Measurement properties of the one-minute sit-to-stand test in children and adolescents with cystic fibrosis: A multicenter randomized cross-over trial
Source: PLoS One. 2021 Feb 12;16(2):e0246781. doi: 10.1371/journal.pone.0246781 (PMC7880481; doi:10.1371/journal.pone.0246781)
Supplement: S4 File — (DOCX) [file pone.0246781.s006.docx]

**Protocole Tests de terrain et Mucoviscidose :**

**Numéro ID-RCB : 2016-A01377-44**

**Numéro CNIL : 2021977 v 0**

**TITRE DU PROJET**

Comparaison du Test de Lever de Chaise (STS-Test) et du Test De Marche de 6 minutes (TDM6) chez des enfants et adolescents atteints de mucoviscidose

**DIRECTEUR DU PROJET**

Centre de Ressource et de Compétences de la Mucoviscidose (CRCM), Hôpital Jacques Monod 76600 Le Havre, dirigé par le Dr Pascal Le Roux, Pneumopédiatre.

**INVESTIGATEURS PRINCIPAUX**

Pascal Le Roux, Pneumopédiatre, Centre de Ressources et de Compétences de la Mucoviscidose, Hôpital Jacques Monod 76600 Le Havre.

pascal.leroux@ch-havre.fr

Yann Combret, Masseur-Kinésithérapeute, Centre de Ressources et de Compétences de la Mucoviscidose, Hôpital Jacques Monod 76600 Le Havre.

yann.combret@gmail.com

Charlotte Gennari, Masseur-Kinésithérapeute, CRCM Caen, Hôpital Côte de Nacre, 14003, Caen.

cha.gennari@gmail.com

Fairuz Boujibar, Masseur-Kinésithérapeute, Centre Hospitalier Universitaire de Rouen, 76000, Rouen

f.boujibar@gmail.com

**Identification de la question**

Le nombre de répétions au Test de Lever de Chaise sur une minute est-il corrélé à la distance parcourue au TDM6 pour des enfants et adolescents atteints de la mucoviscidose ?

**Lieux de la recherche**

Centre de Ressources et de Compétences de la Mucoviscidose, Hôpital Jacques Monod 76600 Le Havre.

Centre de Ressources et de Compétences de la Mucoviscidose, Hôpital Côte de Nacre 14003 Caen.

Centre de Ressources et de Compétences de la Mucoviscidose, Centre Hospitalier Universitaire de Rouen 76000 Rouen.

**OBJECTIFS DE LA RECHERCHE**

**Objectif général**

L’objectif de notre étude est d’évaluer la corrélation entre le Test de Lever de Chaise et le TDM6, et les corrélations entre les résultats de chacun des tests et les paramètres cardio-respiratoires, la qualité de vie et la force des muscles respiratoires et périphériques d’enfants atteints de la mucoviscidose.

**Objectifs spécifiques :**

Objectif principal :

Evaluer la corrélation entre le nombre de répétitions réussies au Test de Lever de Chaise et la distance parcourue au TDM6.

Objectifs secondaires :

- Evaluer les corrélations entre les résultats de chacun des tests et les paramètres cardio-respiratoires suivants : Fréquence cardiaque (FC), Fréquence Respiratoire (FR), Saturation pulsée en O_2_ (SpO_2_), Tension artérielle (TA), Dyspnée et Fatigue musculaire, mesurés avant et après les tests.
- Evaluer les corrélations entre les résultats de chacun des tests et les paramètres suivants mesurés une seule fois avant les tests : VEMS, Capacité Vitale Forcée (CVF), Rapport de VEMS/CVF, Débit Expiratoire de Pointe (DEP), Pressions Inspiratoire (PiMax) et Expiratoire (PeMax) maximales, Force du muscle quadriceps, Qualité de vie et Activité Quotidienne.
- Evaluer les corrélations entre les résultats de chacun des tests et les informations suivantes recueillies à l’inclusion : âge, taille, poids, Indice de Masse Corporelle, Nombre d’exacerbations dans la dernière année écoulée, Nombre d’hospitalisations dans la dernière année écoulée.
- Evaluer les performances du STST pour les enfants les plus jeunes (jusqu’à 10 ans)
- Comparer les valeurs de FC, FR, SpO_2_, TA, Dyspnée et Fatigue musculaire mesurée entre le début et la fin de chaque test séparément.
- Comparer les valeurs de FC, FR, SpO_2_, TA, Dyspnée et Fatigue musculaire entre chacun des deux tests.

**Données à recueillir**

A l’arrivée au CRCM, nous recueillons :

- Age
- Sexe
- Poids
- Taille
- IMC
- Traitements en cours
- Nombre d’exacerbations dans l’année écoulée
- Nombre d’hospitalisations dans l’année écoulée
- Paramètres respiratoires (VEMS, CVF, VEMS/CVF)
- Force des muscles respiratoires (PiMax/PeMax)
- Force du quadriceps
- Qualité de vie
- Consentement du patient

**Au décours des tests de terrain, nous recueillons :**

- Nombre de répétitions au Test de Lever de Chaise
- Distance parcourue au TDM6
- « Travail » du TDM6 (Produit Distance * Poids)
- Fréquence cardiaque
- Fréquence Respiratoire
- Saturation pulsée en O_2_
- Tension artérielle
- Dyspnée
- Fatigue musculaire

**HYPOTHESE DE L’ETUDE**

Nous émettons l’hypothèse que le résultat du Test de Lever de Chaise est corrélé à la distance parcourue au TDM6 pour des enfants atteints de mucoviscidose.

**MOYENS**

Validité externe :

Cette étude permettra d’établir le lien entre le Test de Lever de Chaise et le TDM6. Elle aura également pour but de comparer la tenue des deux tests et d’établir leurs corrélations avec plusieurs marqueurs importants de la mucoviscidose (qualité de vie, activité quotidienne …)

Si les résultats viennent à suivre notre hypothèse, le Test de Lever de Chaise pourrait être utilisé dans l’évaluation des capacités physiques des enfants atteints de mucoviscidose. Si ce test s’avère moins difficile et génère moins de dyspnée ou de stress cardio-respiratoire, celui-ci pourrait constituer une possibilité pour les enfants jeunes ou les patients les plus sévères. Ce test pourrait dans tous les cas s’ajouter à l’ensemble des tests déjà existants pour évaluer la capacité anaérobie et la force des muscles des membres inférieurs de ces jeunes patients.

Ces résultats pourraient alors tenter d’établir la reproductibilité et la validité de ce test et justifieraient la possibilité de rechercher ensuite l’effet d’apprentissage et le MCID (Minimal Clinically Important Difference, la plus petite différence perçue par le patient comme cliniquement importante) pour cette population spécifique afin d’en faciliter l’utilisation.

Validité interne :

Biais de sélection : Tous les enfants des différents Centres seront inclus sur la base des mêmes critères transmis au préalable à chacun des Centres avant le début de l’étude. Tous les enfants atteints de mucoviscidose seront recrutés à une distance d’au moins 4 semaines d’une éventuelle exacerbation. Une exacerbation correspond ainsi à l’utilisation d’une cure d’antibiothérapie intraveineuse [1]. Tous les résultats obtenus par notre analyse auront été réalisés sur la même population de patients nous permettant ainsi d’établir des corrélations entre des profils de patients similaires. En raison du caractère ponctuel de nos mesures aucun perdu de vue n’est à envisager.

Biais d’observation : Les différentes mesures et tests seront réalisées dans les mêmes conditions pour tous les patients. Tous les tests seront réalisés en accord avec les dernières recommandations relatives aux tests de terrain de l’American Thoracic Society et de l’European Respiratory Society [2]. Le même temps de repos de 30 minutes entre les différents tests sera utilisé pour tous les patients inclus [2]. Cela permettra que les tests n’influent pas l’un sur l’autre. Afin de limiter l’effet d’apprentissage connu du TDM6 et du Test de Lever de Chaise, chacun des tests seront réalisés deux fois [2, 3]. Le premier test sera un test d’échauffement à l’issu duquel aucune valeur ne sera utilisée pour l’analyse des résultats. Les encouragements prodigués au patient au cours du TDM6 seront également standardisés entre tous les centres pour éviter qu’ils n’impactent sur les résultats [2]. Bien qu’il n’existe pas de recommandation précise sur les encouragements dans le Test de Lever de Chaise, ceux-ci seront également standardisés entre tous les centres (cf. Déroulement de l’étude). Les tests seront tous effectués au cours de la même demi-journée. Le patient ne devra pas avoir consommé de repas trop copieux moins de 2 heures avant le déroulement du premier test pour ne pas influer sur la mesure. L’ordre des tests sera randomisé.

Biais de confusion : Cette étude est une recherche croisée et randomisée. Les deux tests sont réalisés sur les mêmes patients donc les résultats seront parfaitement comparables. Afin de ne pas modifier la tenue des tests, un ou deux opérateurs maximums par centre seront chargés de la réalisation de ces tests. Les résultats des deux premiers tests préliminaires ne seront pas communiqués au patient afin de ne pas impacter sur les tests réalisés ensuite et comptabilisés pour l’analyse.

**POPULATION DE L’ETUDE**

**Critères d’inclusion :**

- Patients atteints de mucoviscidose ;
- Age de 6 à 18 ans ;
- Garçon ou fille ;
- Consentement libre et éclairé, écrit, par les enfants et les parents après un délai de pré-inclusion de 24h minimum ;
- Suivi au CRCM du Havre, de Caen ou de Rouen
- Enfant à distance (4 semaines minimum) d’une hospitalisation ou d’une antibiothérapie intra-veineuse

**Critères de non-inclusion :**

- Contre-indications cardio-vasculaires à la réalisation des tests de terrain
- Affections musculo-squelettique, neuromusculaires interférents avec la marche (fracture, entorses graves, pathologies du système nerveux périphérique ou central…) ;
- Pathologie pulmonaire restrictive (scoliose …) ou obstructive (asthme) associée ;
- Présence d’un trouble justifiant l’impossibilité de réalisation d’un test de terrain ;
- Absence de compréhension des consignes ;
- Signes cliniques d’infection respiratoire débutante (fébricule, encombrement majoré …)

**Critères d’exclusion :**

- Incapacité à finir le test lors de la mesure
- Survenue d’un des critères de non inclusion

**Nombre de sujets nécessaires**

Afin d’établir une corrélation statistique au moins modérée, nous décidons de fixer le coefficient de corrélation « r » à 0,45. Nous choisissons un risque de première espèce alpha<0,05 et une puissance de l’étude à 80%. Dans ces conditions, un total de 36 patients sera nécessaire pour rejeter l’hypothèse nulle considérant que les tests ne sont pas corrélés. Afin de prévenir un risque éventuel de perte de données ou d’impossibilité de terminer les mesures, nous inclurons 40 patients au final (soit 10% de marge).

**Randomisation des tests**

La randomisation des tests sera réalisée par voie informatique sur une base d’un ratio de 1 : 1 par une tierce personne non impliquée dans l’étude. Elle sera transmise aux investigateurs des différents centres par l’investigateur principal de l’étude (YC).

**Schéma du plan de recherche**

Recueil des premières données

Après obtention de ce consentement, les premières données seront recueillies (âge, sexe, poids, taille, IMC, nombre d’exacerbations et d’hospitalisations dans l’année, résultats de la dernière épreuve fonctionnelle respiratoire : CVF, VEMS, VEMS/CVF).

Une mesure de la force des muscles respiratoires devra ensuite être réalisée. Une mesure des Pressions Inspiratoire et Expiratoire Maximales est alors réalisée à l’aide d’un manomètre électronique MicroRPM PUMA© et d’après la méthode décrite par les recommandations de l’American Thoracic Society [4]. Chaque mesure est réalisée à 3 reprises et seule la meilleure valeur est conservée. Le mode de recueil des données sera le même dans tous les centres.

La force du quadriceps sera ensuite évaluée à l’aide d’un dynamomètre. La dynamométrie a montré son intérêt dans l’obtention de valeurs objectives et reproductibles dans la mucoviscidose [5]. Toutefois, pour des raisons pratiques et budgétaires nous ne pourrons utiliser un dynamomètre isocinétique comme dans l’étude précitée. Nous utiliserons le dynamomètre manuel MicroFET2© qui a montré sa reproductibilité chez l’adolescent sain mais aussi dans un cadre pathologique chez les patients BPCO [6, 7]. Celui-ci montre également une bonne reproductibilité inter-examinateur qui nous permettra d’assurer des mesures comparables entre tous les centres. Nous utiliserons des conditions de mesures standardisées de dynamométrie manuelle « stabilisée » afin de limiter les erreurs de mesures [6, 7]. Chaque enfant sera installé en position assise, jambes pendantes et sanglée avec la hanche et le genou à 90° et le dynamomètre placé à la face antérieur de la jambe. Au total, 5 contractions tenues entre 4 et 6s leur seront demandées. La meilleure des 3 dernières mesures sera comptabilisée pour l’analyse. Un temps de repos de 30 à 60s sera laissé à chaque patient entre deux mesures [6].

Enfin, chaque enfant réalisera une mesure de la qualité de vie réalisée via l’échelle CFQ-R (Cystic Fibrosis Questionnaire) validée dans la mucoviscidose. Cette échelle d’origine française créée par Henry et son équipe et validée ensuite dans de nombreuses autres langues a été validée pour l’enfant de 8 à 16 ans [8]. Nous en utiliserons 3 déclinaisons : pour les enfants de 6 à 11 ans, de 12 et 13 ans et de 14 ans et plus. Ce questionnaire sera rempli de façon standardisée selon les conditions décrites par l’échelle elle-même. L’investigateur ne devra intervenir que pour le remplissage de la grille des enfants de 6 à 11 ans en lisant les questions et en donnant un exemple inaugural. Les cotations seront remplies verbalement par l’enfant et retranscrites de façon objective par l’investigateur. Deux combinaisons de réponses seront possibles selon les questions : « Très vrai / Vrai dans l’ensemble / Un peu vrai / Pas du tout vrai » et « Toujours / Souvent / parfois / Jamais ». Une version du questionnaire sera remplie par les parents pour les enfants de moins de 14 ans. Pour les enfants plus vieux, la grille leur sera distribuée et le remplissage sera assuré par les enfants eux-mêmes. Le questionnaire devra alors être rempli au calme, sans intervention de l’investigateur afin de ne pas fausser la mesure.

Déroulement des tests de terrain

La réalisation des tests de terrain se fera en deux temps. Après le recueil des premières données, chaque test (TDM6 et Test de Lever de Chaise) sera d’abord réalisé une fois. Ces deux premières mesures ne seront pas comptabilisées et n’auront pour but que de limiter l’effet d’apprentissage de ces deux tests. Le patient ne sera pas tenu au courant de son score ou du caractère préliminaire de la mesure.

Après la réalisation de ces deux tests préliminaires, chaque test sera réalisé une seconde fois et cette mesure sera cette fois-ci celle utilisée dans notre analyse des résultats. Un temps de repos de 30min sera octroyé aux patients entre les tests préliminaires et le premier test comptabilisé. Des mesures de la Fréquence Cardiaque, de la Fréquence Respiratoire, de la Saturation pulsée en O_2_, de la Tension artérielle, de la dyspnée et de la fatigue musculaire (échelle de Borg modifiée) devront être réalisées avant et après chacun des tests.

*Réalisation du Test de Lever de Chaise*

Le Test de Lever de Chaise sera réalisé dans un environnement calme et similaire pour tous les patients d’un même centre. Compte tenu de la variabilité de l’âge et donc de la taille des patients, nous ne pouvons utiliser le critère de hauteur de la chaise (taille standard 46cm) comme standardisation. Mais afin de réaliser les tests dans les mêmes conditions, les patients devront tous être installés avec la hanche et le genou à 90° de flexion et ce indépendamment de la hauteur de la chaise. Le test sera au préalable réalisé en démonstration par l’un des investigateurs (toujours le même dans chaque centre) avant le test « d’échauffement ». Il ne sera pas nécessaire de le remontrer avant les mesures réelles puisque le patient en aura déjà réalisé un par lui-même. Les patients devront donc se tenir assis, genou à 90° et les mains sur les hanches pour ne pas utiliser la force des membres supérieurs [9]. Le principe du test leur sera expliqué de la façon suivante : « Nous allons tester ensemble la force de tes jambes. Le but de ce test est de se lever et de se rasseoir le plus de fois possible en une minute. A partir de mon signal, tu devras donc te lever complètement puis te rasseoir complètement le plus de fois possible jusqu’à la fin du test. Je compte mentalement le nombre de répétitions. Je te dirais quand il te restera 15 secondes. Tu as le droit de faire une pause ou de t’arrêter si cela devient trop difficile ». Comme énoncé ci-dessus, les enfants pourront faire une pause ou s’arrêter autant que cela sera nécessaire. Toutefois en cas de pause, le chronomètre ne sera pas arrêté. Le Test de Lever de Chaise durera donc 1 minute et le nombre de répétitions sera comptabilisé manuellement.

*Test de Marche de 6 minutes*

Le TDM6 sera réalisé conformément aux recommandations de l’ATS/ERS [2]. Les conditions du test devront être totalement standardisées entre les centres. Il sera demandé au patient de parcourir la plus grande distance possible en 6 minutes. Le résultat du test utilisé dans l’analyse ultérieure sera la distance parcourue au cours des 6 minutes. Le test sera réalisé dans un couloir droit, peu fréquenté d’au moins 30 mètres de long. Un marquage visible parle patient sera placé aux deux extrémités de la ligne de 30 mètres. Le patient sera encouragé de façon standardisée, par le même opérateur toutes les 60 secondes par des consignes du type « Continue comme ça » tout en lui rappelant le temps restant avant la fin des 6 minutes. Comme pour le Test de Lever de Chaise, le sujet est autorisé à s’arrêter. Mais de nouveau, le temps n’est pas arrêté et chaque évènement de ce type devra être noté.

*Repos*

Un temps de repos incompressible de 30 minutes devra être respecté entre les tests « d’acclimatation » et le premier test comptabilisé dans l’analyse. De même, un second temps de repos de 30 minutes également devra être observé entre le premier et le second test. Ce temps de repos devra être réalisé dans un environnement calme, propice à la récupération. Un rafraichissement pourra être proposé au patient. L’ingestion de boissons sucrées sera à éviter pour ne pas interférer avec la tenue du second test. Il sera demandé au patient de ne pas faire d’effort durant cette période.

Schéma résumé du design de l’étude

Enfant atteint de la mucoviscidose répondant aux critères d’inclusion

*Délai de pré-inclusion (24H minimum)*

Consentement libre et écrit

Recueil des premières données

Randomisation des tests

TDM6

Test de Lever de Chaise

Test de Lever de Chaise

TDM6

Analyse des données

Cette recherche sera conduite conformément au protocole, aux dispositions législatives et réglementaires en vigueur.

**Plan de recherche**

Début des inclusions : Septembre 2017

Durée d’inclusion estimée : 2 ans

**Analyse**

Les analyses statistiques seront effectuées à l’aide du logiciel GraphPad Prism 5. La distribution des variables sera évaluée par le test de Shapiro-Wilk. Les tests paramétriques et non paramétriques adaptés seront utilisés en conséquence. Nous utiliserons les tests de Pearson ou de Spearman pour les corrélations et les tests T de Student pour données appariées pour comparer les résultats pré et post tests et les résultats inter-tests.

Le seuil de significativité statistique est placé à 0,05.

**Surveillance et critères d’arrêt**

Tous les investigateurs impliqués dans la réalisation des tests sont des professionnels de santé habilités et habitués à la pratique de ces tests. Les enfants seront constamment sous la surveillance des personnes impliquées dans la recherche dans tous les centres. Nous n’envisageons aucun évènement indésirable important.

Tous les examens seront arrêtés immédiatement à la demande de l’enfant ou de son/ses représentants légaux ou à l’apparition d’un critère d’exclusion.

**Droits d’accès aux documents :**

Seules les personnes qui dirigent la recherche auront accès aux formulaires de recueil de données.

Les enfants ou leurs parents pourront avoir accès à leur formulaire de recueil de données à leur demande à tout moment.

**Conservation des données :**

Les données seront envoyées à M. Yann Combret, investigateur principal, au CRCM du Havre après recueil dans chaque centre.

Les données seront conservées dans un placard particulier fermant à clé, dans les locaux du CRCM du Havre, dans un local fermant lui-même à clé.

**Rapport final :**

Nous espérons publier cette recherche dans une revue médicale de langue anglo-saxonne, indexée dans la base de données anglophone « Pubmed ».

**BIBLIOGRAPHIE**

[1] O’Neill K, Tunney MM, Johnston E, Rowan S, Downey DG, Rendall J et al. Lung clearance index in adults and children with cystic fibrosis. *Chest*. 2016; [EPub] DOI 10.1016 /j. chest.2016.06.029.

[2] Holland AE, Spruit MA, Troosters T, Puhan MA, Pepin V, Saey D et al. An official European Respiratory Society/American Thoracic Society technical standard: field walking tests in chronic respiratory disease. *Eur Respir J*. 2014; 44: 1428-1446.

[3] Ziegler B, Rovedder PME, Oliveira CL, Silva FA, Dalcin PTR. Repeatability of the 6-minute Walk test in adolescents and adults with cystic fibrosis. *Respir care*. 2010; 55(8): 1020-1025.

[4] American Thoracic Society/European Respiratory Society. ATS/ERS Statement on respiratory muscle testing. *Am J Respir Crit Care Med*. 2002; 166(4): 518-624.

[5] Hussey J, Gormley J, Leen G, Greally G. Peripheral muscle strength in young males with cystic fibrosis. *J Cyst Fibros*. 2002; 1(3): 116-21.

[6] Mentiplay BF, Perraton LG, Bower KJ, Adair B, Pua YH, Williams GP et al. Assessment of lower limb muscle strength and power using hand-held and fixed dynamometry: a reliability and validity study. *PLoS One*. 2015 ; 10(10) : e0140822.

[7] Bachasson D, Villiot-Danger E, Verges S, Hayot M, Perez T, Chambellan A et al. [Maximal isometric voluntary quadriceps strength assessment in COPD]. *Rev Mal Respir.* 2014; 31(8): 765-70.

[8] Bohannon RW, Kindig J, Sabo G. Isometric knee extension force measured using a hand-held dynamometer with or without belt stabilization. *Physiother Theory Pract*. 2012; 28: 562-8.

[9] Henry B, Aussage P, Grosskopf C, Goehrs JM. Development of the Cystic Fibrosis Questionnaire (CFQ) for assessing quality of life in pediatric and adult patients. *Quality of life research*. 2003; 12: 63-76.

[10] Radtke T, Puhan MA, Hebestreit H, Kriemler S. The 1-min sit-to-stand test – A simple functional capacity test in cystic fibrosis? *J Cyst Fibros*. 2016; 15(2): 223-6.
