# Supplementary material for: Measurement properties of the one-minute sit-to-stand test in children and adolescents with cystic fibrosis: A multicenter randomized cross-over trial
Source: PLoS One. 2021 Feb 12;16(2):e0246781. doi: 10.1371/journal.pone.0246781 (PMC7880481; doi:10.1371/journal.pone.0246781)
Supplement: Attachment — Submitted filename: renamed_86099.docx [file pone.0246781.s007.docx]

**COMMENTS – Manuscript PONE-D-20-11696**

Title: “Validation of the one-minute sit-to-stand test to measure functional exercise capacity in children and adolescents with cystic fibrosis: a multicenter randomized cross-over trial”

**Important note: This review pertains only to ‘statistical aspects’ of the study and so** **‘****clinical aspects’ [like medical importance, relevance of the study,** **‘clinical significance and implication(s)’ of the whole study, etc.] are to be evaluated [should be assessed] separately/independently. Further please note that any ‘statistical review’ is generally done under the assumption that (such) study specific methodological [as well as execution] issues are perfectly taken care of by the investigator(s). This review is not an exception to that and so does not cover clinical aspects {however, seldom comments are made only if those issues are intimately / scientifically related & intermingle with ‘statistical aspects’ of the study}.**

**COMMENTS:**

I believe that the value of ‘r’ reported in line 73 is Pearson’s Correlation coefficient [(r=0.49; p<0.01)]. In the light of the following note on Assessing Pearson’s Correlation coefficient*, conclusion given in line 76 [that ‘The STST may not be used as a complete alternative to the 6MWT’] is highly appreciated.

* Statistical test usually used to assess significance of Pearson’s ‘Correlation coefficient (r)’ is ‘t’ [where t = { r √ [(n-2) / (1-r^2^)] }for df=n-2, n is sample size] and here Ho is that the population/standard value of ‘r’ is zero. You need r=0.878 to be significant at 5% but you need r=0.273 if n=50 & you need only r=0.088 if n=500. ‘P-value’ heavily depends on sample size. Therefore, it is customary to conclude/interpret as a ‘Very strong positive correlation/association/relationship only if r = 0.70 or higher.

As you can see the ‘r’ [(r=0.49; p<0.01)] value here indicates a positive association/correlation/relationship which is not very strong though highly ‘significant’ statistically.

Further note that Pearson’s Correlation coefficient is not a measure of ‘Agreement/Concordance’ [instead ‘Kappa Coefficient’ (for categorical data) or ‘intra-class correlation coefficient ICC’ (for numerical/continuous data) are generally used for assessing/measuring Agreement/Concordance].

All these things are mentioned here because [according to lines 165-67: The sample size estimation showed that 36 participants were necessary to detect a correlation coefficient of 0.45 between the STST and 6MWT performances, with 80% power and an alpha level of 0.05] sample size estimation is done on the basis of detection of a correlation coefficient. Intra class correlation coefficient (ICC) were used to verify reliability [lines 182-84: Intra class correlation coefficient (ICC) were calculated using a random effects model for coherence of mean measures to verify reliability between the first and the second test for both STST and 6MWT] and not for assessing/measuring Agreement/Concordance between STST and 6MWT. Nevertheless, what is done is perfectly alright since Intra class correlation coefficient (ICC) is also a good measure of ‘reliability’.

A minor suggestion: In Table 2 [**Table 2. Pre- and Post-exercise test cardio-respiratory response comparisons for the STST and 6MWT in children with CF**] good that both ‘Pre- and Post-exercise’ values are given (essential as description), however comparison could have been done on ‘change scores’. I think that would have been more appropriate. Is not that?

I do not agree with account given in lines 307-318 that a strong correlation was found between STST and 6MWT in the 14 youngest children (≤10 years) just because r=0.60 {for above reasons}. I do not think that ‘this correlation may support the use of the STST as a first approach to functional exercise capacity assessment in children ≤10 years’. However, [as pointed out in ‘Important note’ in the beginning that] this review pertains only to ‘statistical aspects’ of the study and so ‘clinical aspects’ should be assessed separately.

Further please note that any ‘statistical review’ is generally done under the assumption that (such) study specific methodological [as well as execution] issues are perfectly taken care of by the investigator(s). This review is not an exception.
