# Supplementary material for: Measurement properties of the one-minute sit-to-stand test in children and adolescents with cystic fibrosis: A multicenter randomized cross-over trial
Source: PLoS One. 2021 Feb 12;16(2):e0246781. doi: 10.1371/journal.pone.0246781 (PMC7880481; doi:10.1371/journal.pone.0246781)
Supplement: Attachment — Submitted filename: Response to Reviewers.docx [file pone.0246781.s009.docx]

From: Yann Combret, 27^th^ November 2020

To: Dr. Andrea Martinuzzi

Academic Editor, Plos One

Dear Dr. Martinuzzi,

Thank you for your invitation to provide a revised version of our work. We are sincerely grateful to you and the reviewers for the time you spent reviewing our paper entitled *“Measurement properties of the one-minute sit-to-stand test in children and adolescents with cystic fibrosis: a multicenter randomized cross-over trial”*. We are pleased that the reviewers appreciated the corrections made after their suggestions. The other aspects outlined by the Reviewer#2 have been addressed in this revised version. The following is a point-by-point response to the reviewers’ comments. We have provided a marked-up version of our manuscript and a clean version along with this response according to your recommendations. We hope that you will find the responses and revisions satisfactory and that the manuscript now meets *Plos One* publication criteria.

We look forward to hearing from you in the near future.

Yours sincerely.

Yann COMBRET and Gregory REYCHLER

*Please find below our point-by-point response to the reviewers (with modifications highlighted).*

**Response to Reviewer #2**

*The authors have provided sufficient justification to my queries and have addressed most of my concerns. The manuscript improved substantially. However, I would like to suggest them to review some other aspects.*

Thank you for this positive comment and your helpful suggestions. The following is a point-by-point response to your new comments.

*1- Line 200, Page 200: To investigate criterion validity, it is recommended to use tests that have the same characteristics and objective, and ideally are the gold standard. The authors should specify which outcomes were used to investigate criterion validity.*

Thank you for this comment. Criterion validity of the STST to measure functional exercise capacity was measured though the correlation between STST performance and the 6MWD. The gold standard for exercise testing in CF patients is indeed laboratory exercise testing using cycle or treadmill CPET; but, the 6MWT is the most widely used field test worldwide and should be considered whenever CPET is unachievable *(Hebestreit H, Arets HGM, Aurora P, Boas S, Cerny F, Hulzebos EHJ, et al. Statement on Exercise Testing in Cystic Fibrosis. Respiration. 2015;90: 332–351)*.

The measurement undertaken was precised at the end of the ‘Introduction’ (see Line 103) and is now further described in the ‘Methods’ according to the reviewer comment.

P12, L201: “Criterion validity of the STST to measure functional exercise capacity was explored by measuring the correlation between STST performance and the 6MWD derived from the reference test performance (i.e. 6MWT), using Spearman or Pearson correlation coefficients in the complete study sample, in children ≤10 years of age and in children aged 11-18 years.”

*2- Although learning effect and intra-rater reliability was performed, they were not presented in study’s conclusion. Since validity has not been stablished, the authors should describe these results at the conclusion to support the use of 1STST.*

Thank you for this suggestion. The ‘Conclusion’ has been rephrased to encompass these results.

P23, L394: “The simplicity of the STST for both children and practitioners, and the very good intra-rater reliability reported in this study, makes it a reasonable second option when 6MWT or CPET cannot be performed, but healthcare providers should be aware that these tests cannot be used interchangeably. Nonetheless, as both tests revealed a significant learning effect that should be taken to account, the STST could be helpful to reduce the duration of exercise testing.”

*3- Although moderate correlations were found for both analysis with the entire sample and with children≤10 years of age, the result with children≤10 years seems to have higher correlation. Was there a correlation with the sample of children>10 years age? The average age of the sample is 12 years, perhaps the characteristics of the sample have influenced the results. The authors should discuss the result in the correlation analysis with children<10 years age.*

Thank you for these suggestions. The correlation between STST and 6MWD was added for children >10 years of age in the ‘Results’ section of the manuscript. As the reviewer will notice, the correlation is significant; but, lower than the one of younger children (r=0.43; p=0.04). Indeed, as the reviewer hypothesized, the characteristics of the subgroups could be responsible for this difference. Hence, both STST and 6MWT performance are linked with sex; and the sex ratio was different between both subgroups *(Reychler G, Cabillic M, Morales Mestre N, Poncin W, Audag N, Caty G. Predictive model for the 1-minute sit-to-stand test in healthy children aged 6 to 12 years. Ann Phys Rehabil Med. 2020; Li AM, Yin J, Au JT, So HK, Tsang T, Wong E, et al. Standard Reference for the Six-Minute-Walk Test in Healthy Children Aged 7 to 16 Years. Am J Respir Crit Care Med. 2007;176: 174–180).* There was 64% of boys in the ‘younger children’ group, and 23% of boys in the ‘older children’ group. One hypothesis is that in the ‘younger children’ group, the sex ratio was almost equivalent, leading to more heterogenous performances which could have strengthened the correlation between both tests *(Swinscow TDV, Campbell MJ. Statistics at square two. 10., [upgraded] ed. London: BMJ; 2002).* On the opposite, the unbalanced ratio in the ‘older children’ group could have decreased the variability among performances, thereby lowering the correlation coefficient calculated. 6MWD was also more heterogenous in the ‘younger children group’ (i.e. higher SD), probably because this group was smaller (14 children vs 22 in the ‘older group’). Notwithstanding, the difference in correlation coefficient is small, and both correlations could be labeled as ‘moderate’ and this result requires confirmation from larger cohorts.

P20, L314: “Finally, a small trend for a higher correlation between STST and 6MWT performance in children ≤ 10 years of age, compared to older children (>10 years of age) was observed in this study (r=0.54 vs r=0.43, respectively). Interestingly, a sex effect on both STST and 6MWT performance has previously been described; and the sex ratio was unequally distributed between those subgroups (64 vs 23% of boys between the children ≤ 10 years of age and >10 years of age, respectively) [32,33]. A higher heterogeneity in both tests performances in the group of children ≤ 10 years of age could thereby have led to a higher correlation coefficient [18]. Therefore, the STST could be a promising tool for exercise testing in young children with CF, but confirmation in a larger sample of participants is needed.”

*4- In line with item 3, to add information to the reader, the authors could describe in table 1 and table 2 the characteristics of the children≤10 years age.*

Thank you for this helpful proposal. The characteristics of the children ≤ 10 years of age (and subsequently those of the children aged 11-18 years) are now detailed in Table 1 and 2.
